# Melanism in *Peromyscus* Is Caused by Independent Mutations in *Agouti*

**DOI:** 10.1371/journal.pone.0006435

**Published:** 2009-07-30

**Authors:** Evan P. Kingsley, Marie Manceau, Christopher D. Wiley, Hopi E. Hoekstra

**Affiliations:** 1 Department of Organismic and Evolutionary Biology and the Museum of Comparative Zoology, Harvard University, Cambridge, Massachusetts, United States of America; 2 Department of Biological Chemistry, School of Medicine, University of California Irvine, Irvine, California, United States of America; University of Chicago, United States of America

## Abstract

Identifying the molecular basis of phenotypes that have evolved independently can provide insight into the ways genetic and developmental constraints influence the maintenance of phenotypic diversity. Melanic (darkly pigmented) phenotypes in mammals provide a potent system in which to study the genetic basis of naturally occurring mutant phenotypes because melanism occurs in many mammals, and the mammalian pigmentation pathway is well understood. Spontaneous alleles of a few key pigmentation loci are known to cause melanism in domestic or laboratory populations of mammals, but in natural populations, mutations at one gene, the *melanocortin-1 receptor* (*Mc1r*), have been implicated in the vast majority of cases, possibly due to its minimal pleiotropic effects. To investigate whether mutations in this or other genes cause melanism in the wild, we investigated the genetic basis of melanism in the rodent genus *Peromyscus*, in which melanic mice have been reported in several populations. We focused on two genes known to cause melanism in other taxa, *Mc1r* and its antagonist, the agouti signaling protein (*Agouti*). While variation in the *Mc1r* coding region does not correlate with melanism in any population, in a New Hampshire population, we find that a 125-kb deletion, which includes the upstream regulatory region and exons 1 and 2 of *Agouti*, results in a loss of *Agouti* expression and is perfectly associated with melanic color. In a second population from Alaska, we find that a premature stop codon in exon 3 of *Agouti* is associated with a similar melanic phenotype. These results show that melanism has evolved independently in these populations through mutations in the same gene, and suggest that melanism produced by mutations in genes other than *Mc1r* may be more common than previously thought.

## Introduction

From complex patterns, like the stripes of a tiger, to the simple changes in the presence/absence of pigment, as in arctic hares, the diversity in mammalian pigmentation is vast [1]. But in addition to diversity among species, there is often appreciable variation in pigmentation within species. Because members of the same species that differ in their pigmentation phenotype can be crossed, this within-species variation is amenable to traditional genetic analyses. In addition, the molecular genetic factors that regulate mammalian pigmentation are relatively well known [reviewed in 2]–[4], thus enabling the genetic bases of these phenotypes to be explored. Furthermore, a nontrivial advantage to studying pigmentation traits is that variation is often easily detectable by eye. Mutant phenotypes that affect the coloration of the entire body are especially conspicuous and are easily recognized by both captive breeders and field biologists. One such phenotype is dark pigmentation or melanism. It is clear that melanism has evolved many times in wide variety of animal taxa [5].

The genes that can cause darkening of coat color have been studied most thoroughly in the laboratory mouse. Although experimentally induced mutations in over 25 genes can produce dark fur in lab mice [6], spontaneous coat-darkening mutations have been reported in only four genes: the *Agouti* signaling protein (*Agouti*), attractin (*Atrn*), melanocortin-1 receptor (*Mc1r*), and mahogunin (*Mgrn*) [7]–[10]. The protein products of three of these genes, *Mc1r, Agouti*, and *Atrn*, interact at the surface of pigment-producing cells (melanocytes) and constitute the machinery responsible for “pigment type switching,” the ability of melanocytes to switch between the production of dark brown/black (eumelanin) and light yellow/red pigment (pheomelanin). *Mc1r* is a membrane-bound receptor that, when active, signals the melanocyte to produce eumelanin, whereas *Agouti* is a paracrine signaling protein that antagonizes *Mc1r*, causing the melanocyte to produce pheomelanin. Thus, mutations that cause either constitutive- or hyper-activation of *Mc1r* or loss-of-function of *Agouti* will result in a melanic phenotype. The functions of *Atrn* and *Mgrn* are not as well understood, although *Atrn* is thought to stabilize interactions between *Mc1r* and *Agouti* [11]. Here, we focus on *Mc1r* and *Agouti* because their interaction has been well characterized in the lab mouse and thus can be extended to the study of melanism in other taxa.

Melanic phenotypes have evolved both in nature and in captivity in a wide diversity of animals and in some cases their genetic basis has been identified. In captive vertebrates, spontaneous mutants of *Agouti*, *Mc1r, Mgrn*, and *Atrn* have all been found to cause melanism [e.g.], [12], [13], [10,8]. In natural populations, however, mutations in *Mc1r* are most commonly associated with melanism [e.g. 12], [14]–[16], although both *Agouti* and *Atrn* are larger mutational targets. In addition, mutations in *Agouti* or *Atrn* that reduce protein expression or activity lead to melanism; these knock-out mutations are certainly more likely to occur than the gain-of-function *Mc1r* mutations that cause melanism because there are more ways to “break” a gene than to “improve” a gene's activity. Thus, it is unclear why *Mc1r* has repeatedly been shown to be associated with melanism in nature and a key question is: are melanism-inducing mutations in *Agouti* not found because they occur less often, or are they simply more difficult to detect?

**Figure 1:**
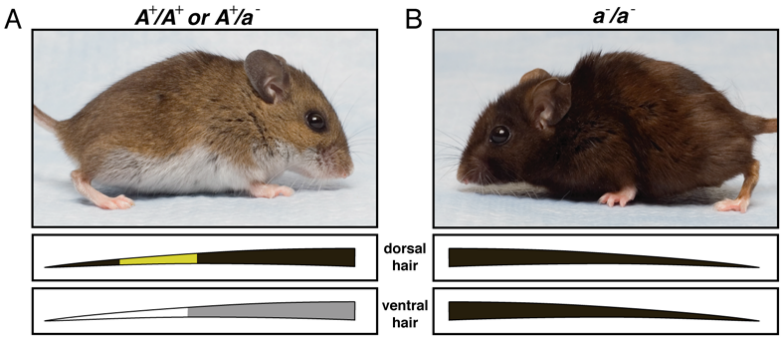
Pigmentation phenotypes of *P. maniculatus*. (A) Typical wild type individual, dorsal hairs are banded (containing both pheomelanin and eumelanin) and ventral hairs are white with a light grey base. This phenotype is dominant to the melanic phenotype. (B) Melanic individual with completely eumelanic hairs. These mice were captured in Hubbard Brook Experimental Forest, NH.

To address this question, we studied melanism in the deer mouse, *Peromyscus maniculatus* (Figure 1). Melanism has been reported in several populations of *Peromyscus*; melanic individuals have been captured in a number of locations in North America, including New Hampshire [17], California [18], Michigan [P. Myers, pers. comm.], and Alaska [C. Conroy, pers. comm.]. Although it is unclear if these melanic phenotypes affect fitness, their repeated occurrence provides us with multiple comparisons of the same phenotype in the same genetic system (i.e. species). Horner et al. [17] showed that, in mice from New Hampshire, melanism is caused by a recessive allele at a single locus. The authors suggested the locus might be *Agouti*, based on its similarity to the nonagouti phenotype in *Mus*. Here we uncover the molecular variation that causes melanism in *P. maniculatus* from New Hampshire and show that the *Agouti* gene is responsible. We also investigate the molecular basis of melanic phenotypes from geographically distant populations of *P. maniculatus* and find that melanism has independently arisen at least three times and by different mutations in the same gene, *Agouti*, in two of those cases.

## Results

### Melanism caused by a single, recessive locus

The inheritance of the melanic phenotype in the New Hampshire strain of *P. maniculatus* was previously investigated by Horner et al. [17]. We confirmed their results with two crosses that clearly demonstrate that a single autosomal recessive allele is responsible for the melanic phenotype (Table S1).

### Agouti is a candidate gene for Peromyscus melanism

**Figure 2:**
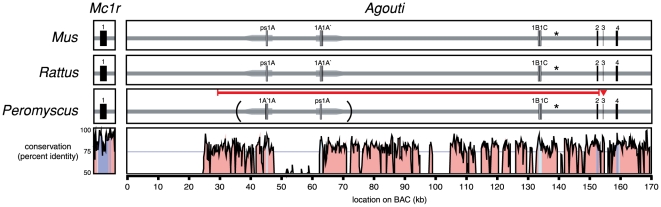
Schematic and VISTA alignment of the *Mc1r* and *Agouti* loci in *Mus, Rattus*, and *Peromyscus*. Dark blocks represent coding sequences; light blocks represent untranslated exons. *Mc1r* consists of a single exon that spans approximately 1.5 kb similar to its *Mus* ortholog. The *Agouti* locus spans over 100 kb. Grey arrows indicate a duplication present in all three taxa; brackets indicate the inversion of the duplicated region in *Peromyscus*. Asterisks mark the location of a conserved region that is necessary for Agouti expression (Y. Chen and G. S. Barsh, pers. comm.). The red line and red arrowhead mark the locations of the *a^Δ125kb^* deletion and the *a^Q65term^* premature stop codon, respectively. The conservation plot was generated by aligning *Peromyscus* BAC sequence and sequence from the *Rattus* genome using LAGAN [75] and plotting conservation with mVISTA [76].

The phenotypic similarity between melanic *Peromyscus* and mouse (*Mus*) *Agouti* mutants and the recessive nature of the melanic allele in *P. maniculatus* suggested that *Agouti* is a strong candidate gene. We sequenced a 180 kb BAC clone containing *Agouti* from *P. maniculatus rufinus* and compared it to the corresponding sequence from the *Mus* genome. In *Mus*, the *Agouti* gene consists of four non-coding exons (1A, 1A′, 1B, and 1C) and three protein-coding exons (2, 3, and 4); this arrangement appears to be conserved in other mammals, including rat (*Rattus*). Sequences orthologous to the exons in *Mus* and *Rattus* are conserved in the *P. maniculatus* sequence (Figure 2). However, when compared to the published genome sequences of *Mus* and *Rattus*, an inversion of the region containing exons 1A and 1A' is present in *P. maniculatus*. Inversions in this region are sometimes associated with differences in ventral pigmentation in different strains of *Mus* [19].

To determine whether a mutation(s) in the *Agouti* locus is associated with melanism, we genotyped the 49 offspring of an *A^+^/a^−^*×*A^+^/a^−^* cross. We found a perfect association between successful amplification of exon 2 and phenotype: we always produced an exon 2 product of the expected size in wild type individuals (*A^+^/−*, N = 34) but never in melanic (*a^−^/a^−^*, N = 15) individuals. In addition, while we amplified all the *Agouti* exons (untranslated 1A, 1A', 1B, 1C and translated 2–4) in all wild type offspring, we were able to amplify only exons 3 and 4 from melanic mice. By contrast, we did not find any amino acid differences between wild type and melanic individuals in the entire *Mc1r* coding region. These results strongly suggest, first, that melanism is caused by variation at the *Agouti* locus and second, that a large deletion in *Agouti* may be responsible for the melanic phenotype.

### Large deletion in Agouti associated with melanism

To determine if there was a deletion in the *a^−^* allele and if so, its size, we used genome-walking PCR to sequence upstream (5′) of exon 3. We found that sequence identity between the wild type BAC sequence and the melanic *Agouti* allele extends about 1.3 kb 5′ of exon 3. Thereafter, the melanic *Agouti* allele sequence is identical to the sequence 125 kb upstream in the wild type BAC (Figure 2). Thus, melanic *P. maniculatus* are homozygous for an allele with a large 125 kb deletion (*a^Δ125kb^*), which eliminates the main regulatory region, the noncoding exons 1A, 1A', 1B, 1C, and coding exon 2.

**Figure 3:**
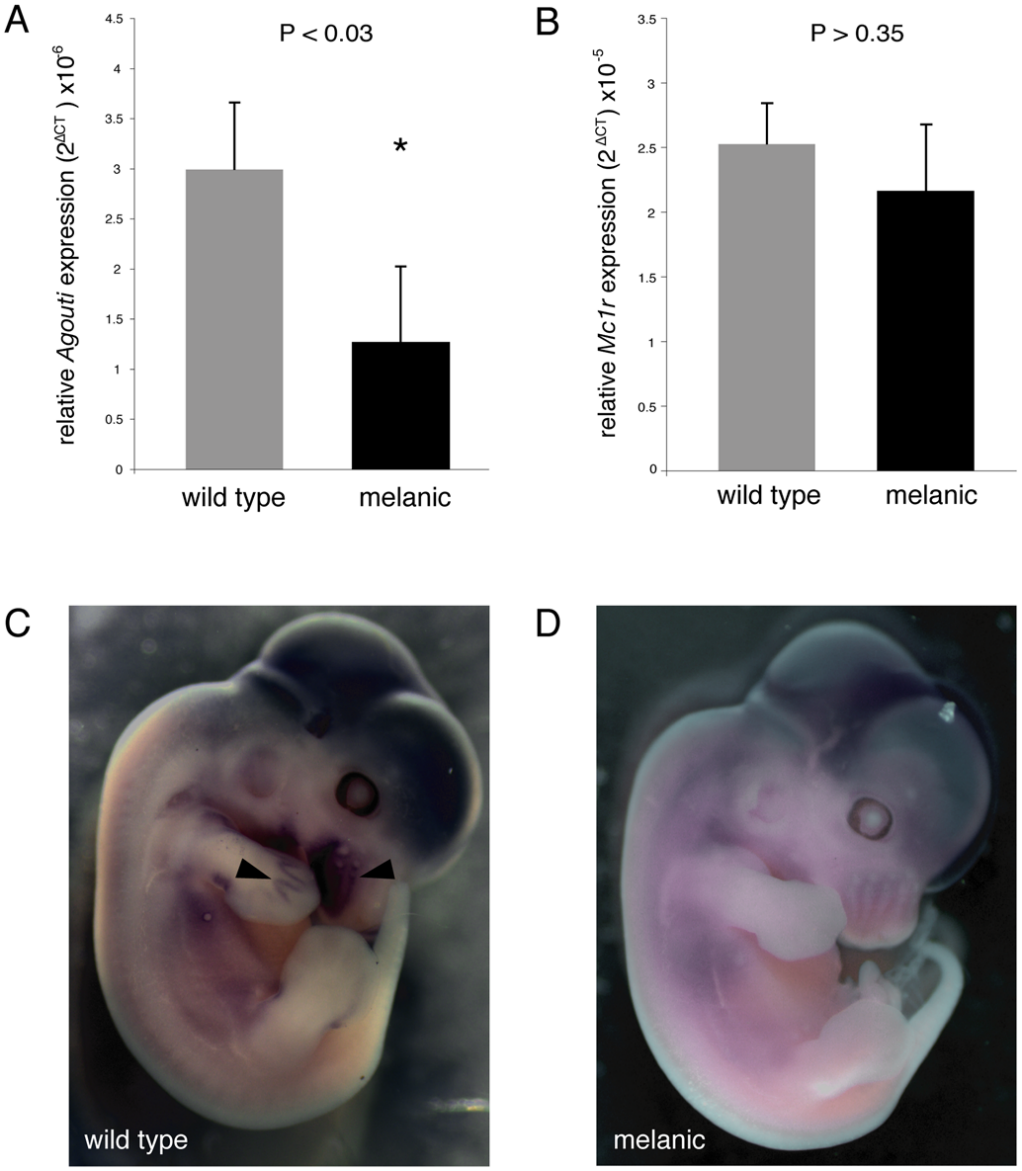
*Agouti* and *Mc1r* expression in wild type and melanic mice. (A, B) Relative expression of *Agouti* and *Mc1r* transcripts in dorsal skin of P4 *P. maniculatus* was measured by quantitative RT-PCR. Expression level of the target gene is standardized with that of *β-actin*. We compared relative expression levels of each gene with Student's t-test (two-tailed, unequal variance). For each phenotype class, N = 5. (A) *Agouti* expression is significantly higher in the dorsal skin of wild type mice than in melanic mice; expression level in melanic mice is not significantly different from zero. (B) *Mc1r* expression in wild type and melanic mice does not significantly differ. Bars indicate standard error. (C,D) Lateral views of whole-mount *in situ* hybridizations for Agouti in E12.5 embryos. (C) Wild type embryos express *Agouti* in the whisker plate and the limbs (arrows). (D) *Agouti* expression is not detected in *a^Δ125kb^* homozygote embryos.

To test whether this 125 kb deletion affects the abundance of *Agouti* transcript, we measured *Agouti* mRNA in the skin of P4 pups. In animals heterozygous for the wild type and the *a^Δ125kb^* alleles, levels of *Agouti* expression were significantly higher than those of animals homozygous for *a^Δ125kb^* (Figure 3A). These data show that the *a^Δ125kb^* allele produces significantly less *Agouti* mRNA transcript and is thus likely the cause of melanism. *Mc1r* transcript levels, on the other hand, were not significantly different between melanic and wild type individuals (Figure 3B). In addition, we performed *in situ* hybridizations on 12.5 day-old embryos to determine whether *Agouti* is expressed in melanic embryos. At this stage, wild type embryos express *Agouti* in the whisker plate and in parts of the limbs (Figure 3C), an expression pattern similar to that seen in *Mus* [20]. We did not detect any Agouti expression in melanic embryos (Figure 3D).

### Molecular basis of melanism in Alaskan mice

**Figure 4:**
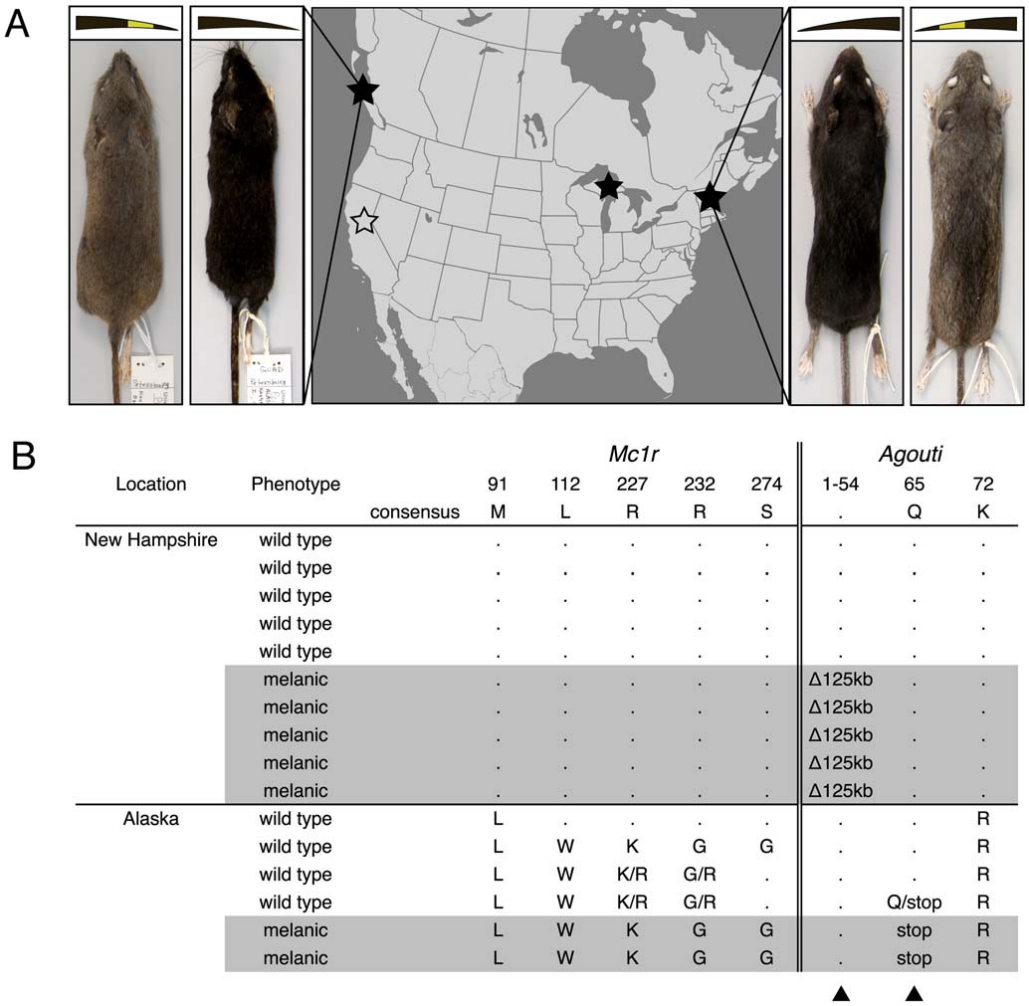
Melanism evolved multiple times independently in *P. maniculatus*, twice by mutations in the *Agouti* gene. (A) Wild type and melanic museum skins from Shrubby Island, AK (C. Conroy, pers. comm.) and Hubbard Brook Experimental Forest, NH [17]. Illustrations of the dorsal hair pattern are shown above each specimen. Black stars represent locales included in this study; white star denotes another location where melanic *Peromyscus* were reported [18]. (B) Table of polymorphism for *Mc1r* and *Agouti* coding sequences. Arrows indicate two sites harboring mutations that are perfectly correlated with melanism.

To determine if the same gene and same mutation was responsible for melanism in other populations of *P. maniculatus*, we sequenced both *Mc1r* and *Agouti* in melanic and wild type mice from an additional population. First, we sequenced *Mc1r* in melanic (N = 2) and non-melanic (N = 4) *P. maniculatus* from Alaska and found four amino acid polymorphisms segregating in the sample (Figure 4). None of these polymorphisms likely cause the melanic phenotype for several reasons: (1) none of these mutations overlaps with any previously described darkening mutations, (2) all four amino acids appear in other, non-melanic individuals from other populations of *P. maniculatus* (Figure 4), and (3) none of the polymorphisms correlate with the melanic phenotype in this population.

In the same sample, we also sequenced the coding exons of *Agouti* and found one segregating amino acid polymorphism, a mutation at nucleotide position 193 (in exon 3) that results in a change from glutamine to a stop codon at amino acid position 65 (*a^Q65term^*). This premature stop codon eliminates exon 4, which contains a cysteine-rich region that is integral to the function of the *Agouti* protein (Figure 4; [21], [22]). Thus, this mutation very likely results in a non-functional protein. Individuals both homozygous and heterozygous for the *a^Q65term^* allele had the wild type phenotype, consistent with the *a^Q65term^* allele being recessive and its being a null allele. Though the small number of animals sampled does not allow us to rule out the involvement of other loci, these data strongly suggest that the *a^Q65term^* allele is the cause of the melanic phenotype in the Alaskan population.

Melanism also has been reported in a third population, *P. m. gracilis* from the upper peninsula of Michigan [P. Myers, pers. comm.]. We sequenced the complete coding regions of *Agouti* in a single melanic individual. The *Agouti* sequence possesses neither the *a^Q65term^* nor the *a^Δ125kb^* mutation, nor does it contain any obvious melanism-causing mutations in *Mc1r*, demonstrating a third independent origin of melanism in *P. maniculatus*.

## Discussion

The results of our laboratory crosses confirmed that melanism in New Hampshire *P. maniculatus* is caused by a single, recessive allele. In laboratory mice, dominant melanism is usually caused by alleles of *Mc1r*, while recessive melanism is usually caused by alleles of *Agouti*. Consistent with this dominance hierarchy, we found that melanism in *P. maniculatus* is perfectly correlated with the presence of an allele (*a^Δ125kb^*) with a large deletion at the *Agouti* locus. When mice are homozygous for this allele, the abundance of *Agouti* transcript in the skin is significantly lower than that in individuals with a single copy of the wild type *Agouti* allele. This accords with the observation that the deleted region contains the 5′ untranslated regions that are important for temporal and spatial regulation of *Agouti* and probably any associated *cis*-regulatory information. The deletion also encompasses exon 2, which contains the start of the *Agouti* protein (amino acids 1–54). Together, this evidence strongly suggests that the *a^Δ125kb^* allele causes melanism in *P. maniculatus* from New Hampshire.

Sequencing of *Agouti* and *Mc1r* coding regions in melanic individuals from other geographic locations shows that melanism arose independently at least three times in *P. maniculatus*. Melanic individuals from Shrubby Island, AK are homozygous for an allele (*a^Q65term^*) of *Agouti* that contains a premature stop codon in exon 3. This mutation is predicted to result in a non-functional protein. Although we cannot rule out contributions of linked variation to the melanic phenotypes possessed by mice from New Hampshire and Alaska, given the likely effects of the *Δ125kb* and *Q65term* mutations and the known effects of null *Agouti* alleles in other taxa, it is very likely that these mutations represent the causative variation underlying these melanic phenotypes. The melanic individual from Michigan possesses neither the *a^Δ125kb^* allele nor the *a^Q65term^* allele; melanism in this population must be caused either by variation at another locus or possibly by unexamined variation at the *Agouti* or *Mc1r* loci.

This study presents two cases in which a specific molecular variant at the *Agouti* locus appears to cause melanism in a natural population. *Mc1r* mutants represent the vast majority of cases of melanism in natural populations of mammals, despite many occurrences of melanic *Agouti* mutants in captive and domestic stocks (Table 1). There are a number of possible explanations for this discrepancy.

**Table 1:** Spontaneous alleles causing melanic phenotypes in mammals and birds.

gene	wild/domestic	taxon	allele name	homozygous phenotype	mutation	reference
*Agouti*	d	*Canis familiaris*	a	black coat	R96C	[55]
	d	*Coturnix japonica*	Y*RB	black coat	8 bp deletion (frameshift)	[56]
	w/d	*Equus caballus*	A^a^	black coat	11 bp deletion in exon 2 (frameshift)	[57], [58]
	d	*Felis catus*	ASIP-Δ2	black coat	2 bp deletion in exon 2 (frameshift)	[12]
	d	*Mus*	a^22R^	black coat	F118S	[59]
	d	*Mus*	a	black coat	11 kb insertion in intron 1	[7]
	d	*Ovis aries*	A^a^	black coat	unknown non-coding mutation	[60]
	d	*Rattus*	a	black coat	19 bp deletion in exon 2 (frameshift)	[61]
	d	*Vulpes vulpes*	a	dark “silver” coat	166 bp deletion of entire exon 2	[62]
*Mahogunin*	d	*Mus*	Mgrn1^md^	dark brown coat	5 kb insertion in intron 11	[10]
	d	*Mus*	Mgrn1^md-2J^	dark brown coat	5 kb insertion in exon 12	[10]
	d	*Mus*	Mgrn1^md-5J^	dark brown coat	8 kb insertion in intron 2	[10]
*Attractin*	d	*Mus*	Atrn^mg^	dark brown coat	5 kb insertion in intron 26	[63]
	d	*Mus*	Atrn^mg-L^	dark brown coat	5 kb insertion in intron 27	[63]
	d	*Mus*	Atrn^mg-3J^	dark brown coat	5 bp deletion in exon 16 (frameshift)	[8]
	d	*Mus*	Atrn^mg-6J^	dark brown coat	large deletion of N-terminal exons	[64]
*β-defensin 103*	w/d	*Canis familiaris Canis lupus*	K^B^	black coat	1 bp deletion (frameshift)	[65], [66]
*Mc1r*	d	*Bos taurus*	E^D^	black coat	L99P	[67]
	d	*Coturnix japonica*	E	dark brown plumage	E92K	[13]
	d	*Gallus gallus*	E	black plumage	E92K	[68], [69]
	d	*Mus*	E^so^	dark brown coat	L96P	[9]
	d	*Mus*	E^so-3J^	dark brown coat	E92K	[9]
	d	*Ovis aries*	E^D^	black coat	M73K, D119N*	[70], [71]
	d	*Sus scrofa*	E^D1^	black coat	L99P and D121N	[72]
	d	*Vulpes vulpes*	E^A^	dark “silver” coat	C125R	[62]
	w	*Alopex lagopus*	blue	dark grey/blue coat	G5C, F280C	[73]
	w	*Anser c. caerulescens*	blue	dark plumage	V85M	[14]
	w	*Chaetodipus intermedius*	dark	dark brown coat	R18C, R109W, R160W, Q233H*	[15]
	w	*Coereba flaveola*	GSV	black coat	E92K	[16]
	w	*Herpailuris yaguarondi*	MC1R-Δ24	dark brown coat	24bp deletion	[12]
	w	*Mus*	E^tob^	black coat until 8 weeks	S69L	[9]
	w	*Panthera onca*	MC1R-Δ15	black coat	15 bp deletion	[12]
	w	*Stercorarius parasiticus*	dark	dark plumage	R230H	[14]
	w	*Sula sula*	dark	dark plumage	V85M and H207R	[74]

* indicates mutations in complete linkage disequilibrium.

One possible explanation involves dominance. Haldane [23] suggested that, when natural selection acts on new (i.e., rare) beneficial mutations, adaptation will be biased toward fixing dominant alleles, which are immediately visible to selection (but see [24]). Thus, we expect that when melanism is adaptive, we may see a prevalence of melanic *Mc1r* mutants. On the other hand, if melanism is deleterious and is being held at mutation-selection equilibrium, we might expect melanism caused by mutations in *Agouti* if they are recessive. Thus, depending on environmental conditions, expectations regarding the fixation probabilities of *Mc1r* versus *Agouti* alleles are different. In *Peromyscus*, the melanic alleles in both populations described in this study were found at low frequencies – 3–7% assuming Hardy-Weinberg equilibrium ([17]; data not shown) – and there is no obvious association between melanism and environmental conditions as observed in other species (e.g., pocket mice; [25]), suggesting these alleles may not be adaptive. Thus, if melanic phenotypes are often fixed from new dominant mutations rather than standing genetic variation, this may explain the prevalence of melanism caused by *Mc1r*.

Second, if mutations in *Agouti* have greater negative pleiotropic effects than mutations in *Mc1r*, then we would expect to see more evolution in the latter. Having fewer negative pleiotropic consequences of mutations at a locus translates to less evolutionary constraint (or higher net selection coefficients). While deleterious effects may be tolerated when organisms are raised in captivity, they could have important fitness consequences in nature. Whether differing amounts of pleiotropy of mutations at these loci affects the evolution of melanism is difficult to say, because mutations in both *Agouti* and *Mc1r* may affect traits other than pigmentation. Mutations in *Mc1r*, for example, have recently been discovered to have effects in the nervous system [26]. Pleiotropy is especially well documented in *Agouti*: ectopic expression of *Agouti* in *Mus* can result in obesity and lethality [27], [28] and null mutants in *Rattus* and *Peromyscus* exhibit behavioral differences [29], [30]. But pleiotropic consequences may be mitigated by the precise type and location of mutations. It has been predicted that for any given gene, mutations in the *cis*-regulatory elements may minimize antagonist pleiotropic effects relative to those in coding regions because such mutations can alter the time or place of gene expression in some tissues while preserving gene function in others [31]–[33]. Our data provide examples of mutations that are associated with morphological diversity: in one case, a premature stop codon, and in a second, a large deletion of both regulatory and exonic DNA. Thus, our data show, despite potential pleiotropic effects, both *cis*-regulatory and coding mutations in a highly pleiotropic gene, *Agouti*, cause a visible melanic phenotype that segregates in natural populations. Alternatively, it is possible that the melanic alleles in this study do generate negative pleiotropic effects that prevent them from increasing in frequency.

The third possibility is that a bias exists toward detecting mutations in the small *Mc1r* locus versus the larger, more complex *Agouti* locus. In fact, one would expect that there are more possible mutations that can cause a null *Agouti* allele than a constitutively active *Mc1r* allele. Many cases of melanism that have not yet been assigned a precise mutational cause (e.g., some populations of pocket mice [34]; pocket gophers [35]; leaf warblers [36]) may be caused by variation at *Agouti*, or indeed other loci.

Understanding the genetic basis of phenotypes that have arisen independently underpins studies of convergence by natural selection. While the fitness consequences of the melanic phenotypes in this study are unknown, studies of pigmentation may be uniquely positioned to identify convergence and to uncover its molecular basis because pigmentation traits are easily recognizable and many of the genes involved in producing pigments are well characterized. As the number of cases of convergence on a particular phenotype increases, so does our understanding of the constraints limiting the ways that phenotypes can evolve. In some cases, like stomach lysozyme [37], [38], pelvic reduction in sticklebacks [39], [40], or cyclodiene resistance in a number of insect taxa (reviewed in [41]), evolution appears to be tightly constrained, and the same gene is the repeated target of natural selection. In other cases, such as pigmentation, many different genetic mechansims can produce the same phenotype (beach mice [42], [43]; pocket mice [34]; *Drosophila* [44]; cavefish [45], [46]; *Heliconius* [47]). However, in these cases and others, it seems that a handful of proteins at key regulatory points in the pigmentation pathway are major targets of evolution change (e.g., *Mc1r*/*Agouti* in vertebrates; *ebony*/*yellow* in Drosophila; *DFR* in flowering plants [48]) Thus, natural selection may repeatedly target either the same key points in a genetic pathway or even the same genes to produce the most beneficial phenotype while minimizing deleterious pleiotropy. Future work on additional phenotypes in additional taxa will shed light on the myriad ways that evolution can generate morphological diversity.

## Materials and Methods

### Ethics statement

Experiments were approved by the Harvard University Institutional Animal Care and Use Committee and were conducted in accordance with National Institutes of Health regulations governing the humane treatment of vertebrate animals.

### Animal samples

For this study, we first focused on mice from a wild-derived captive strain of melanic *Peromyscus* maintained at the Peromyscus Genetic Stock Center (Columbia, South Carolina). These melanic animals (*P. maniculatus gracilis*) are derived from mice captured in 1977 at the Hubbard Brook Experimental Forest in New Hampshire [17]. Second, to study the genetic basis of other melanic phenotypes, we obtained tissue samples of melanic mice from natural history collections originally captured in two additional populations in Alaska (*P. m. keeni*) and Michigan (*P. m. gracilis*).

### Genetic Crosses

To determine the genetic basis of melanism in *P. maniculatus* from New Hampshire, we conducted two types of genetic crosses. First, to confirm dominance, we set up four mating pairs of wild type *P. maniculatus bairdii* and melanic *P. m. gracilis* [17]. Second, for the single-locus test, we established three mating pairs and backcrossed mice that were heterozygous for the melanic allele to the wild type. We then scored the phenotypes of the resulting offspring by eye.

### Tissue Samples

We acquired tissue samples from two additional populations of *P. maniculatus* that harbor melanic individuals. First, we received tissue samples from mice (*P. m. keeni*) inhabiting Shrubby Island in southeastern Alaska (University of Alaska Museum of the North, accession numbers UAM20875, 20876, 20878, 20880, 20882), although the status of *P. m. keeni* as a subspecies of *P. maniculatus* [49] or its sister species, *P. keeni*, [50] is unresolved. We also acquired a tissue sample of a single melanic individual of *P. m. gracilis* from Macinac County, Michigan (University of Michigan Museum of Zoology). Tissue samples from another melanic population (*P. m. gambeli*) in California [18] were not available.

### PCR amplification and sequencing

We extracted genomic DNA from liver using the DNeasy kit (Qiagen, Valencia, CA). Primers and PCR conditions used to amplify the complete *Agouti* coding exons are shown in Table S2; these amplification primers were also used in the sequencing reactions. Primers to amplify the *Mc1r* coding region were used as previously described [51]. We used ABI3730xl and 3130xl sequencers (Applied Biosystems, Foster City, CA) and aligned all sequences in Sequencher (Gene Codes, Ann Arbor, MI). When a deletion was identified, we used genome-walking to identify the breakpoint (GenomeWalker Universal kit; Clontech, Mountain View, CA); primers are shown in Table S3. Once we identified the precise deletion breakpoint, we designed primers across the deletion to genotype individuals; these primers are listed in Table S2.

### BAC sequencing

To examine the *Mc1r* and *Agouti* loci in *Peromyscus*, we screened an available BAC library for *P. m. rufinus*. For the *Agouti* locus, we captured the entire described regulatory region [52] by using two probes representing untranslated exon 1A/1A' and the last coding region, exon 4, which span approximately 100 kb in *Mus*. A 160 kb BAC containing *Mc1r* and a 180 kb BAC containing *Agouti* were then shotgun sequenced by Agencourt (Beverly, MA) until sequences from each BAC could be assembled into a single contig for each locus and all gaps were filled.

### Real time quantitative PCR

To quantify *Mc1r* and *Agouti* transcript levels in wild type and melanic mice from New Hampshire, we used quantitative real-time PCR to detect *Mc1r* and *Agouti* mRNA in the skin of 4-day-old (P4) pups, a time when *Agouti* expression is high [52]. First, we extracted total RNA from dorsal skin that had been frozen in liquid nitrogen with an RNeasy kit (Qiagen). Next, we generated cDNA pools by reverse transcribing from ∼1ug total RNA with Superscript II reverse transcriptase and poly-dT_(20)_ primer. Finally, we measured transcript abundances with TaqMan custom probe based on exon-4 sequence (Applied Biosystems, Foster City, CA) as previously described [53] on a Mastercycler Realplex2 (Eppendorf North America, New York, NY). We compared expression of the target transcript to that of *β-actin* by calculating 2^ΔCT^ in which ΔCT is the difference between the target and *β-actin* CTs for a given sample. We assayed expression level for each individual in duplicate.

### *In situ* hybridization

We generated a cDNA pool from *Peromyscus* embryonic skin at E13, and amplified the entire coding region of *Agouti* (exons 2 to 4). An *Agouti* anti-sense riboprobe was obtained by RNA synthesis reaction and used to perform *in situ* hybridization on wild type and melanic embryos at E12.5 as previously described [54].

## Supporting Information

Table S1 Melanism is caused by a single autosomal recessive allele in *P. maniculatus*. We found complete recessivity of the melanic phenotype in the New Hampshire strain of P. maniculatus consistent with previous observations [17]. Offspring resulting from crosses between homozygous wild type mice (*A^+^/A^+^*) and homozygous melanic mice (*a^−^/a^−^*) were all phenotypically indistinguishable from wild type (N = 64), confirming that the allele(s) causing the melanic phenotype is recessive to the wild type allele. In a second experiment, offspring that were heterozygous for the melanic allele (*A^+^/a^−^*; although phenotypically wild type) – were intercrossed, resulting in 49 offspring, of which 34 (69%) were the wild type phenotype, 15 (31%) were melanic, and none had an intermediate phenotype. The ratio of phenotypes is not significantly different from 3∶1 (χ^2^ = 0.82, 1 d.f., p>0.35), confirming that a recessive allele at a single locus is responsible for the melanic phenotype in this strain of *P. maniculatus*. Subsequent genotyping of these offspring revealed a ratio of homozygous wild type:heterozygote:homozygote melanic ratio not significantly different from 1∶2∶1 (χ^2^ = 0.88, 2 d.f., p>0.6).
(0.04 MB DOC)

Table S2 Standard PCR primer sequences and conditions
(0.03 MB DOC)

Table S3 Genome walking PCR primer sequences
(0.03 MB DOC)
