# Supplementary material for: Melanism in Peromyscus Is Caused by Independent Mutations in Agouti
Source: PLoS One. 2009 Jul 30;4(7):e6435. doi: 10.1371/journal.pone.0006435 (PMC2713407; doi:10.1371/journal.pone.0006435)
Supplement: Table S1 — Melanism is caused by a single autosomal recessive allele in P. maniculatus. We found complete recessivity of the melanic phenotype in the New Hampshire strain of P. maniculatus consistent with previous observations [17]. Offspring resulting from crosses between homozygous wild type mice (A+/A+) and homozygous melanic mice (a−/a−) were all phenotypically indistinguishable from wild type (N = 64), confirming that the allele(s) causing the melanic phenotype is recessive to the wild type allele. In a second experiment, offspring that were heterozygous for the melanic allele (A+/a−; although phenotypically wild type) – were intercrossed, resulting in 49 offspring, of which 34 (69%) were the wild type phenotype, 15 (31%) were melanic, and none had an intermediate phenotype. The ratio of phenotypes is not significantly different from 3∶1 (χ2 = 0.82, 1 d.f., p>0.35), confirming that a recessive allele at a single locus is responsible for the melanic phenotype in this strain of P. maniculatus. Subsequent genotyping of these offspring revealed a ratio of homozygous wild type:heterozygote:homozygote melanic ratio not significantly different from 1∶2∶1 (χ2 = 0.88, 2 d.f., p>0.6). (0.04 MB DOC) [file pone.0006435.s001.doc]

**Table S1.** Melanism is caused by a single autosomal recessive allele in *P. maniculatus*

| **cross** | **# of wt offspring** | **# of melanic offspring** | **expected ratio** | **χ^2^** | **P-value** |
| --- | --- | --- | --- | --- | --- |
| A^+^/A^+^ x a-/a- | 64 | 0 | --- | --- | --- |
| A^+^/a- x A^+^/a- | 34 | 15 | 3:1 | 0.82 | > 0.35 |

We found complete recessivity of the melanic phenotype in the New Hampshire strain of *P. maniculatus* consistent with previous observations [17]. Offspring resulting from crosses between homozygous wild type mice (*A^+^/A^+^*) and homozygous melanic mice (*a^-^/a^-^*) were all phenotypically indistinguishable from wild type (N = 64; Table 1), confirming that the allele(s) causing the melanic phenotype is recessive to the wild type allele. In a second experiment, offspring that were heterozygous for the melanic allele (*A^+^/a^-^*; although phenotypically wild type) -- were intercrossed, resulting in 49 offspring, of which 34 (69%) were the wild type phenotype, 15 (31%) were melanic, and none had an intermediate phenotype. The ratio of phenotypes is not significantly different from 3:1 (χ^2^=0.82, 1 d.f., p>0.35), confirming that a recessive allele at a single locus is responsible for the melanic phenotype in this strain of *P. maniculatus*. Subsequent genotyping of these offspring revealed a ratio of homozygous wild type:heterozygote:homozygote melanic ratio not significantly different from 1:2:1 (χ^2^=0.88, 2 d.f., p>0.6).
