# Supplementary material for: Melanism in Peromyscus Is Caused by Independent Mutations in Agouti
Source: PLoS One. 2009 Jul 30;4(7):e6435. doi: 10.1371/journal.pone.0006435 (PMC2713407; doi:10.1371/journal.pone.0006435)
Supplement: Table S2 — Standard PCR primer sequences and conditions (0.03 MB DOC) [file pone.0006435.s002.doc]

**Table S2.** Standard PCR primer sequences and conditions

| **primer name** | **sequence** | **region of Agouti amplified** | **annealing temperature (degrees C)** |
| --- | --- | --- | --- |
| AAgoutiS10 | CACATCCTCCTACCACCATC | exon 2 | 55 |
| AAgoutiAS10 | AGGAATGAGCGAGAAAGAGC |
| AAgoutiS9 | CTTCCACTCCGAGACTTCCC | exon 3 | 55 |
| AAgoutiAS9 | TAGGCCAGGAAACCTTTAGG |
| AgE4_F1 | CGAGGTCTCTGGTCCCATAC | exon 4 | 55 |
| AgE4_R3 | CAGGACCAGAGCTCGGTAAA |
| DELspan_F1 | ACGTGGACATTTGGTCAGAG | a^Δ125kb^ deletion breakpoint | 55 |
| DELspan_R1 | CAGGAGATCGCTAGCTAATG |
