# Supplementary material for: Melanism in Peromyscus Is Caused by Independent Mutations in Agouti
Source: PLoS One. 2009 Jul 30;4(7):e6435. doi: 10.1371/journal.pone.0006435 (PMC2713407; doi:10.1371/journal.pone.0006435)
Supplement: Table S3 — Genome walking PCR primer sequences (0.03 MB DOC) [file pone.0006435.s003.doc]

**Table S3.** Genome walking PCR primer sequences

| **primer name** | **sequence** |
| --- | --- |
| AgGWE3_up1 | GCACAAAGAGCAAAGCCAGATGTTTC |
| AgGWE3_up2 | GGAAGTCTCGGAGTGGAAGAGAGAAAG |
| AgGWE3_up3 | GTTAGAGACCTGGACTTGGTCTTTTG |
| AgGWE3_up4 | CTTGATCACCACAAGTACCTTTAACCT |
| AgGWE3_up5 | GTCTCTGGAAGTGGACTCAG |
| AgGWE3_up6 | CAGTTCCTGGCACATCTGTG |
| AgGWE3_up7 | TGCACTTTCAAAGGACCCAG |
| AgGWE3_up8 | CACAAGGTGACTCACAACTATC |
| AgGWE3_up9 | CTTGCCTCTTTTGACTTCTCTG |
| AgGWE3_up10 | GACCACTTTGGCAAACCTC |
| AgGWE3_up11 | CAGGAGATCGCTAGCTAATG |
| AgGWE3_up12 | GCGCTCTATAGGAAAGCAAC |
